# Low tricuspid annular plane systolic excursion is associated with a poor outcome in patients with COVID-19: A systematic review and meta-analysis

**DOI:** 10.1097/MD.0000000000028971

**Published:** 2022-02-25

**Authors:** Ye Tian, Huaihai Lu, Xuefang Liu, Yinlong Zhao, Pei Zhang

**Affiliations:** The Second Hospital of Hebei Medical University, Shijiazhuang, China.

**Keywords:** coronavirus disease 19, echocardiography, meta-analysis, tricuspid annular plane systolic excursion

## Abstract

Background: This systematic review and meta-analysis aimed to assess whether tricuspid annular plane systolic excursion (TAPSE) could be used as a prognostic tool in patients with coronavirus disease 19 (COVID-19).

**Methods:**

Studies on the relationship between TAPSE and COVID-19 since February 2021. Standardized mean difference (SMD) and 95% confidence intervals were used to assess the effect size. The potential for publication bias was assessed using a contour-enhanced funnel plot and Egger test. A meta-regression was performed to assess if the difference in TAPSE between survivors and nonsurvivors was affected by age, sex, hypertension or diabetes.

**Results:**

Sixteen studies comprising 1579 patients were included in this meta-analysis. TAPSE was lower in nonsurvivors (SMD −3.24 (−4.23, −2.26), *P* < .00001; I^2^ = 71%), and a subgroup analysis indicated that TAPSE was also lower in critically ill patients (SMD −3.85 (−5.31, −2.38,), *P* < .00001; I^2^ = 46%). Heterogeneity was also significantly reduced, I^2^ < 50%. Pooled results showed that patients who developed right ventricular dysfunction had lower TAPSE (SMD −5.87 (−7.81, −3.92), *P* = .004; I^2^ = 82%). There was no statistically significant difference in the TAPSE of patients who sustained a cardiac injury vs those who did not (SMD −1.36 (−3.98, 1.26), *P* = .31; I^2^ = 88%). No significant publication bias was detected (*P* = .8147) but the heterogeneity of the included studies was significant. A meta-regression showed that heterogeneity was significantly greater when the incidence of hypertension was <50% (I^2^ = 91%) and that of diabetes was <30% (I^2^ = 85%).

**Conclusion:**

Low TAPSE levels are associated with poor COVID-19 disease outcomes. TAPSE levels are modulated by disease severity, and their prognostic utility may be skewed by pre-existing patient comorbidities.

**Trial retrospectively registered (February 12, 2021):**

PROSPERO CRD42021236731

## 1 Introduction

Coronavirus disease 19 (COVID-19), which is caused by the severe acute respiratory syndrome coronavirus 2 (SARS CoV-2),^[1]^ is a serious global public health challenge. On August 5, 2021, the cumulative number of COVID-19 cases globally surpassed 200 million, just 6 months after reaching 100 million.^[2]^ As a result, the understanding of the disease and its risk factors are key factors for implementing public health policies at present.

COVID-19 induces cytokine release,^[3]^ thereby creating an inflammatory state that can lead to cardiopulmonary injury.^[4–10]^ Endothelial dysfunction induced by shear stress, hypoxia, autoimmune phenomena, viral infections may initiate the process of excess vasoconstriction, inflammation, and uncontrolled cellular growth,^[11–15]^ and lead to pulmonary hypertension.^[13,15]^ In response to an increase in pulmonary vascular resistance by a factor of 5 to 10, the right ventricle (RV) undergoes hypertrophy, chamber dilatation, fat deposition, fibrosis, and metabolic shifts as pulmonary hypertension progresses.^[16–17]^ Right ventricular function (RVEF) is the major determinant of clinical outcomes and survival among patients with pulmonary hypertension.^[17]^

**Figure 1:**
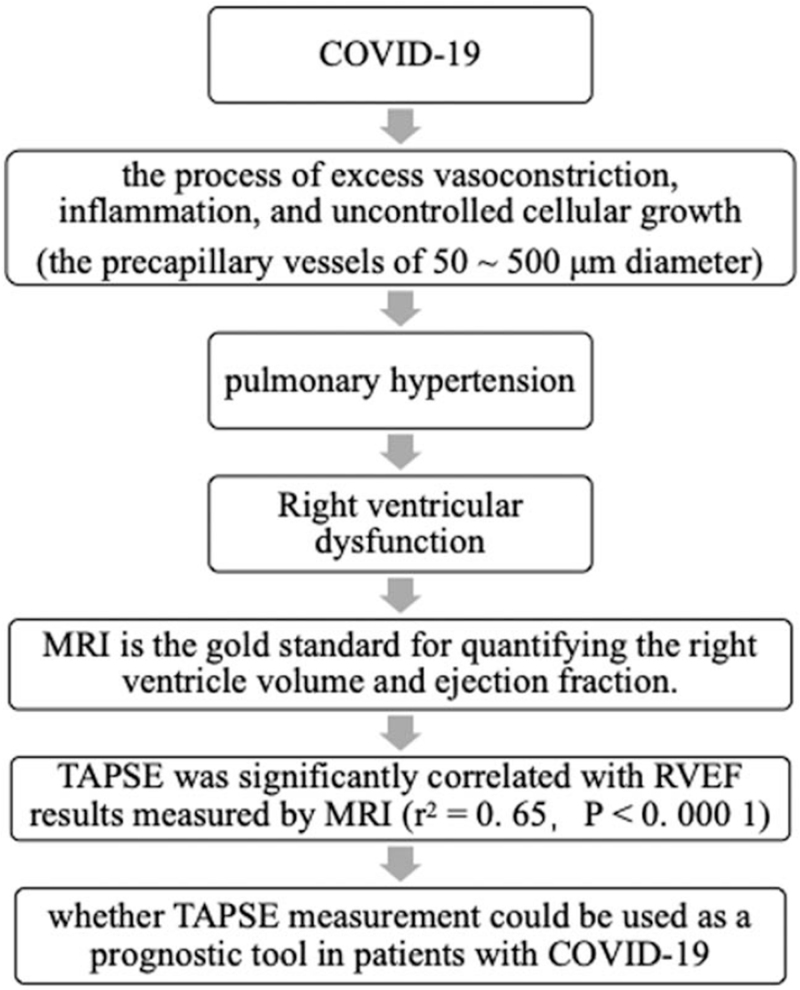
Flowchart. COVID-19 = coronavirus disease 19, MRI = Magnetic resonance imaging, RVEF = right ventricular function, TAPSE = tricuspid annular plane systolic excursion.

Magnetic resonance imaging can accurately assess the function of the RV without being affected by the geometry of the RV, and is the gold standard for quantifying the RV volume and ejection fraction.^[18]^ Hugues et al^[19]^ proved in their study that tricuspid annular plane systolic excursion (TAPSE) was significantly correlated with RVEF results measured by magnetic resonance imaging (*r*^2^ = 0.65, *P* < .0001). TAPSE has also proven to be easy to perform, accurate, reproducible and with little variation between observers. And the American Society of Echocardiography currently recommends using TAPSE measurement as one of the tools to assess RV function.^[20]^ However, diagnostic use of echocardiography during the COVID-19 pandemic is largely based on expert recommendations and lacks data from large samples of evidence-based scientific results (Fig. 1).

Previous studies have shown that TAPSE affects clinical outcomes in patients with COVID-19, but there has been no correlation analysis between TAPSE and RVEF or myocardial injury, nor has the patient's physical condition been considered. The purpose of this systematic review and meta-analysis was to more fully assess whether TAPSE can be used as a prognostic tool for patients with COVID-19 and to provide a reference for the clinical treatment of these patients.

## 2 Methods

A systematic review was performed in accordance with the preferred reporting items for systematic reviews and meta-analyses guidelines (Table S1, Supplemental Digital Content). The protocol for this study was registered in PROSPERO (CRD42021236731).

### 2.1 Eligibility criteria

**Figure 2:**
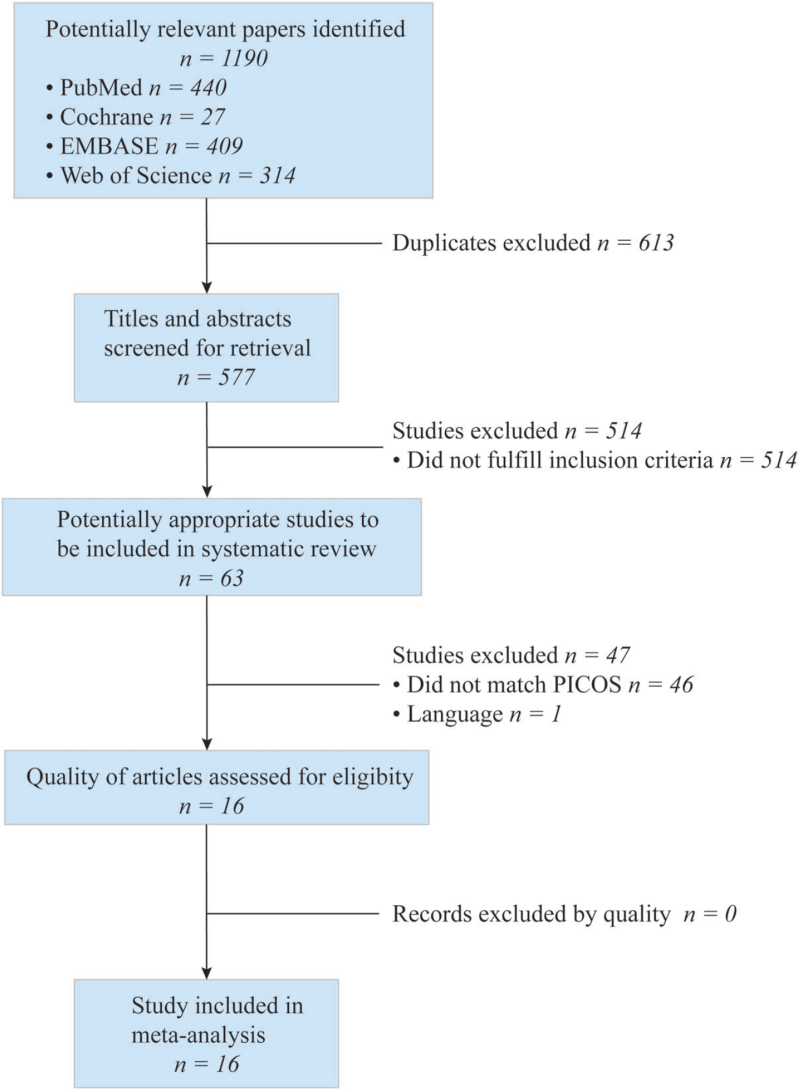
PRISMA flowchart. Participants (P), interventions (I), comparisons (C), outcomes (O), study design (S). PRISMA = preferred reporting items for systematic reviews and meta-analyses.

Eligible articles were identified in the process illustrated in Figure 2.

Inclusion criteria: Studies with primary data on patients with COVID-19 with TAPSE measurements that were used as a prognostic metric. The primary outcome of the work was a “poor outcome,” defined by the authors of the study using some composite of death, disposition to a long-term acute care facility (LTAC), cardiac injury, and right ventricular dysfunction (RVD).

Exclusion criteria: Preprints, review articles, nonresearch letters, commentaries, case reports, and articles not written in English. Preprints were excluded due to inconsistent credibility.

### 2.2 Definitions

The following definitions were used for this analysis: COVID-19 was diagnosed with a positive RT-PCR for SARS CoV-2 with a throat swab, sputum or endotracheal suction sample^[21]^; TAPSE was measured using guidelines set forth by the American Society of Echocardiography.^[22]^ In short, TAPSE was assessed by placing an M-mode cursor through the lateral tricuspid valve annulus in the apical 4-chamber view and measuring the total systolic excursion distance of the tricuspid annulus; Mortality was defined as clinically validated death/nonsurvival^[23]^; LTAC centers are unique institutions that provide a full range of medical services for medically complex patients who require an extended stay in an acute care facility^[24]^; Cardiac injury was defined as an increase in high-sensitivity troponin T or troponin I levels above the 99th percentile upper reference limit in the setting of a newly abnormal electrocardiograph and/or transthoracic echocardiography^[25]^; and RVD was defined by impairment of both TAPSE and S′ so as to reduce false positive classification.

### 2.3 Search strategy and study selection

Articles published as of February 2021 in the PubMed, Cochrane Library, Embase, and Web of Science databases were included. The search strategy used across all databases combined medical subject headings and free words with “AND” and “OR” as the 2 logical operators. Key search terms included “(coronavirus or corona virus) AND Wuhan”, “2019 nCoV”, “2019nCoV”, “2019-nCoV”, “2019 novel coronavirus”, “COVID19”, “COVID 19”, “COVID-19”, “new coronavirus”, “novel coronavirus”, “novel corona virus”, “SARS CoV-2”, or “severe acute respiratory syndrome coronavirus 2” combined with “(cardiac or heart or right) AND ventric∗”, “RV”, “tricuspid annular plane systolic excursion”, or “TAPSE”. Only studies published in English with an available full text were included. When several publications involved the same patient group, the most recent or most complete (largest sample size) study was chosen. Potentially relevant articles were then assessed using our inclusion and exclusion criteria.

### 2.4 Data extraction and quality assessment

Two independent authors extracted data from eligible studies with the help of a standardized data form that included fields for first author, year of publication, country of publication, study design, sample size, age, male sex, hypertension, diabetes, chronic obstructive pulmonary disease, current smoker, outcome (mortality), LTAC disposition, cardiac injury, and RVD.

The quality of each cohort study was evaluated with the Newcastle–Ottawa Scale, and cross-sectional studies were evaluated with the Agency for Healthcare Research and Quality scale. Quality assessments were performed by 2 independent authors. Discrepancies at the end of the assessment were addressed through the inclusion of a third party.

### 2.5 Statistical analysis

The meta-analysis of extracted data was performed using Review Manager 5.4 (Cochrane, London, UK) and STATA 16 (StataCorp, College Station, TX). Standardized mean difference (SMD) and corresponding 95% confidence intervals were estimated with a random-effect meta-analysis. The significance of pooled SMD was evaluated with a *Z*-test, and a *P*-value of <.05 was considered significant. In light of possible sources of heterogeneity, studies were stratified by disease severity. The potential for publication bias was assessed using a contour-enhanced funnel plot and Egger test. A meta-regression was performed to assess if the difference in TAPSE between survivors and nonsurvivors was affected by age, male sex, hypertension or diabetes.

## 3 Results

### 3.1 Baseline characteristics and quality evaluation

The initial search identified 1190 studies. Excluding duplicates, 577 articles were screened with a title and abstract review. The entire manuscript body of 63 relevant articles was assessed, of which 16 met the inclusion criteria for data analysis (Fig. 2). This yielded a total of 1579 patients.^[21,24,26–39]^ Baseline characteristics of the included studies are displayed in Table 1. Of the 10 studies that had mortality as their primary outcome, 4 included critically ill patients. All included studies underwent quality assessment using Newcastle–Ottawa Scale and Agency for Healthcare Research and Quality, and were found to be of sufficient quality to be included in the meta-analysis (Table 1).

**Table 1:** Characteristics and quality assessments of the included studies.

			Experimental group (survivors)					Comparison (nonsurvivors)						
Study	Location	Study design	Patients enrolled	Mean ± SD Age (yr)	Male (n)	Hypertension (n)	Diabetes (n)	Patients enrolled	Mean ± SD age (yr)	Male (n)	Hypertension (n)	Diabetes (n)	Outcome	NOS or AHRQ
Yuji Xie, 2020	China	Prospective cohort	113	61 ± 13	54	–	–	19	64 ± 13	14	–	–	Mortality	8
			92	60 ± 13	43	38	12	40	63 ± 12	25	20	3	Cardiac injury	
Alexander, 2020	UK	Prospective cohort	19	70 ± 3.3	16	12	10	15	75 ± 4	11	6	2	Mortality	9
Dominik, 2020	Germany	Prospective cohort	107	67 ± 15	65	74	25	16	73 ± 16	12	12	5	Mortality	8
Francesca, 2020	Italy	Retrospective cohort	33	63.4 ± 12.7	21	16	5	16	70.5 ± 11.2	10	8	4	Mortality	8
Michele, 2020	Italy	Prospective cohort	69	62 ± 13	53	44	11	25	68 ± 12	17	19	5	Mortality	8
Renuka, 2020	America	Prospective cohort	22	54.9 ± 8.8	11	12	8	30	63.6 ± 12.2	20	24	11	Dead or in LTAC	8
Roya, 2020	Iran	Prospective cohort	75	58.89 ± 15.33	47	9	27	11	58.82 ± 19.92	5	0	2	Mortality	9
François, 2021	France	Prospective cohort	41	56 ± 13.33	33	17	11	26	67 ± 3.25	22	19	13	Mortality	8
Yuman Li, 2020	China	Prospective cohort	102	–	–	–	–	18	–	–	–	–	Mortality	8
Yingxian Liu, 2020	China	Prospective cohort	21	64.1 ± 9.8	7	10	7	22	64.9 ± 10.4	15	9	5	Mortality	9
Stéphanie, 2021	Germany	Prospective cohort	14	53 ± 7.407	11	4	2	18	68 ± 4	17	13	5	Cardiac injury	7
Antonello, 2020	Italy	Prospective cohort	89	55.3 ± 14.5	–	26	–	26	73.5 ± 12.75		16	–	Cardiac injury	7
Hasan, 2020	Turkey	Cross-sectional study	61	–	–	–	–	29	–	–	–	–	Cardiac injury	11
Matteo, 2020	Italy	Cross-sectional study	171	62 ± 5	111	73	31	29	65 ± 5.25	20	11	6	RVD	11
Hani, 2020	UK	Retrospective cohort	54	59 ± 13	40	24	18	20	58 ± 13	18	7	9	RVD	8
Jiwon, 2020	America	Prospective–retrospective cohort	227	65 ± 14	136	145	89	41	66 ± 15	30	29	16	RVD	7

The cohort study used NOS score and the cross section study used AHRQ score. AHRQ = agency for healthcare research and quality, LTAC = long-term acute care facility, NOS = Newcastle–Ottawa Scale, RVD = right ventricular dysfunction, SD = standard deviation.

### 3.2 TAPSE and mortality

**Figure 3:**
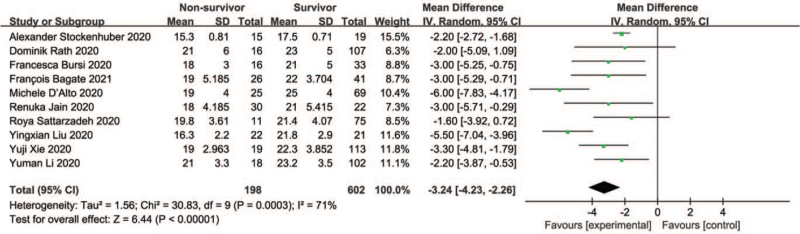
Mean differences between TAPSE of survivors and nonsurvivors. CI = confidence interval, SD = standard deviation.

Changes in TAPSE were reported in 10 trials that involved 602 surviving and 198 nonsurviving patients. TAPSE was lower in nonsurvivors compared with survivors (SMD –3.24 (–4.23, –2.26), *P* < .00001; I^2^ = 71%) (Fig. 3).

**Figure 4:**
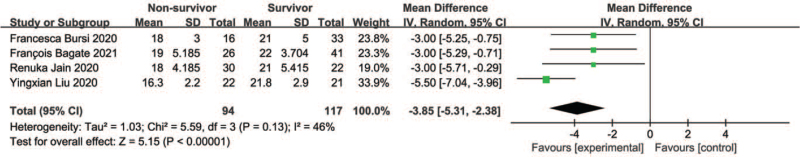
Mean difference in TAPSE of critically ill patients between survivors and nonsurvivors. CI = confidence interval, SD = standard deviation.

Four studies included only critically ill patients. A subgroup analysis showed that TAPSE was also lower in critically ill patients (SMD –3.85 (–5.31, –2.38,), *P* < .00001; I^2^ = 46%) (Fig. 4).

### 3.3 TAPSE and RVD

**Figure 5:**
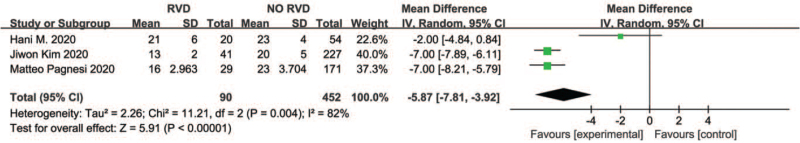
Mean TAPSE difference between patients who went on to develop RVD vs those who did not. CI = confidence interval, RVD = right ventricular dysfunction, SD = standard deviation.

Three trials included the TAPSE of 90 patients with RVD compared with 452 controls. Based on the I^2^ (I^2^ = 82% > 50%) and chi-square test *P*-values (*P* < .05), we chose a random effects model to analyze differences in TAPSE. Pooled results showed that patients with RVD had lower TAPSE (SMD −5.87 (−7.81, −3.92), *P* = .004; I^2^ = 82%) (Fig. 5).

### 3.4 TAPSE and cardiac injury

**Figure 6:**
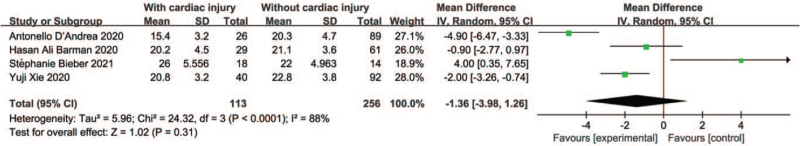
Mean TAPSE difference between patients who sustained a cardiac injury vs those who did not. CI = confidence interval, SD = standard deviation, TAPSE = tricuspid annular plane systolic excursion.

Three trials compared the TAPSE of 113 patients with a cardiac injury to 256 patients with normal cardiac status. There was no statistically significant difference in TAPSE between patients with or without a cardiac injury (SMD –1.36 (–3.98, 1.26), *P* = .31; I^2^ = 88%) (Fig. 6).

### 3.5 Risk of publication bias and relevance

**Figure 7:**
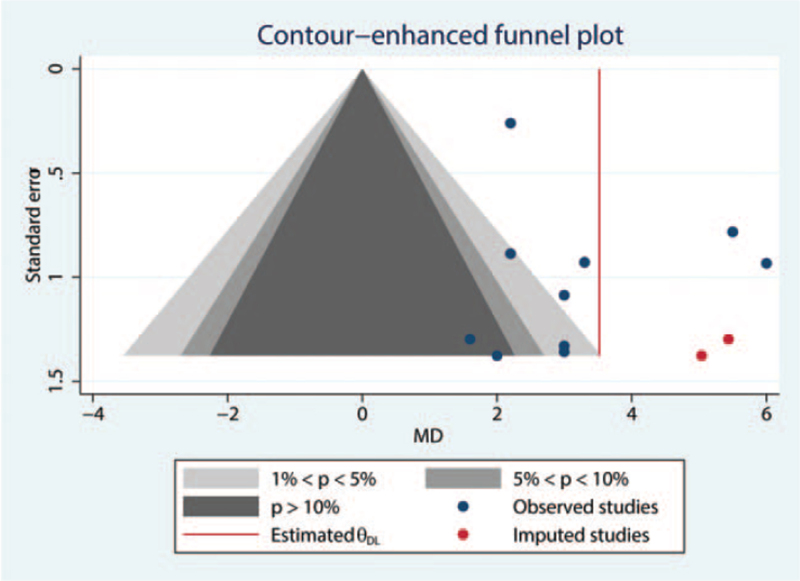
Contour-enhanced funnel plot.

A contour-enhanced funnel plot was symmetric (Fig. 7), which was consistent with the results of Egger test (*P* = .8147) and suggestive of a lack of publication bias. The included studies were therefore deemed comprehensive and yielded statistically reliable results.

### 3.6 Stratified analysis of mortality from COVID-19 infection in patient groups

A meta-regression was performed that found that the difference in TAPSE between nonsurvivors and survivors was significantly affected by hypertension (*P* = .047) and diabetes (*P* = .006), but not age (*P* = .398) or male sex (*P* = .925). A subgroup analysis showed that heterogeneity was significantly greater when the incidence of hypertension was ≥50% (I^2^ = 61%) and that of diabetes was ≥30% (I^2^ = 0%) (Table 2).

**Table 2:** Stratified analysis of mortality from COVID-19 infection in patient groups with different proportions of patients with hypertension or diabetes.

				Heterogeneity	
Variable	No. of studies	SMD (95% CI)	*P*	I^2^	*P*_h_
The prevalence of hypertension					
≥50%	5	−0.78 (−1.16, −0.39)	<.0001	61%	.04
<50%	3	−1.75 (−3.21, −0.28)	.02	91%	<.0001
The prevalence of diabetes					
≥30%	3	−0.57 (−0.87, −0.26)	.0003	0%	.71
<30%	5	−1.44 (−2.21, −0.68)	.0002	85%	<.0001

CI = confidence interval, COVID-19 = coronavirus disease 19, SMD = standardized mean difference.

## 4 Discussion

In a meta-analysis of 16 studies with 1579 patients, lower TAPSE and poor COVID-19 outcomes were independently associated with mortality and RVD, but not cardiac injury. A subgroup analysis showed that critically ill patients had lower TAPSE. Further, a meta-regression analysis showed that hypertension and diabetes had a significant impact on the difference in TAPSE between survivors and nonsurvivors.

Significant heterogeneity was a limitation of the analysis of the cohort and cross-sectional studies included in this study. The first reason for the observed heterogeneity was clinical severity. Our meta-analysis of TAPSE showed that nonsurvivors had lower TAPSE (I^2^ = 71%), whereas our subgroup analysis of critically ill patients indicated that TAPSE (I^2^ = 46%) was equivalent, accompanied by significantly reduced heterogeneity. It may be more applicable in critically ill patients.

The second reason for the observed heterogeneity was that different research centers did not have a standardized protocol for treating patients but rather relied on their respective training and experience. The third reason was that the results of the present work were based on unadjusted estimates. A more precise analysis could be conducted if individual patient data were made available, thereby permitting the assessment of COVID-19 disease severity by age, sex, past medical history, and personal history. A meta-regression demonstrated that differences in TAPSE were affected by hypertension (*P* = .047) and diabetes (*P* = .006), and a subgroup analysis showed that heterogeneity was significantly reduced when the incidence of hypertension was ≥50% or the incidence of diabetes was ≥30%. The fourth reason is that TAPSE may be angle-dependent when the image is off-center due to the enlargement of the RV, and may also be affected by cardiac displacement. Hsiao et al^[40]^ also found the correlation between severe tricuspid regurgitation affecting TAPSE and RVEF in their study, and the effect of severe tricuspid regurgitation should be considered comprehensively when applying TAPSE to evaluate RVEF.

COVID-19-induced systemic inflammation can lead to ARDS, pulmonary hypertension, myocardial injury, and ultimately RV failure. Studies have found that among hospitalized non-Intensive Care Unit patients with COVID-19, pulmonary hypertension was associated with signs of more severe COVID-19 and with worse inhospital clinical outcome.^[35]^ With the increase of pulmonary artery (PA) pressure (right cardiac afterload), stroke volume decreased nonlinearly. TAPSE, which can be used to evaluate RV function, has been proved as an independent prognostic marker of specific pathologies including ARDS.^[39]^ Fauvel et al^[41]^ also reported TAPSE as a good marker of RV contractility, with relative load-independence in the setting of pulmonary hypertension. However, the use of TAPSE as a prognostic marker remains controversial. Shadi et al^[42]^ did not find a correlation between severe sepsis or septic shock and TAPSE. This may have been owing to various factors such as patient comorbidities.

The important role of the RV in the management of the critically ill is increasingly acknowledged, and many novel measures of RV function have been developed. For example, RV free wall longitudinal strain overcomes some of the limitations of conventional diagnosis of RV systolic function parameters, but both lack standardized data and consensus.^[43]^ And some measurement methods are good, such as strains or 3D-derived measures, but they need better image quality and special software. On the other hand, most TAPSE measurements take less than 30 seconds, with high operability and repeatability, TAPSE seems to be more attractive to critically ill patients or primary hospitals.

It has also been suggested that the ratio of TAPSE to PA systolic pressure (PASP) has a stronger association with outcome in COVID-19 patients than TAPSE.^[44]^ With developing pulmonary hypertension, the RV responds to a significantly elevated afterload by increasing its contractility. This response may be accompanied by an increased TAPSE. However, with persistent and progressive pulmonary hypertension, TAPSE may not correspond to an increase in PA pressure.^[45]^ Schmeisser et al^[46]^ also reported that combining TAPSE with PASP did not improve the noninvasive right ventricular (RV) to PA coupling information. Therefore, it seems more meaningful to evaluate RV and PASP separately.

Prior studies have suggested that the cytokine profile of secondary hemophagocytic lymphohistiocytosis is related to COVID-19 severity.^[47]^ Studies by Zhou et al^[1]^ found that patients with cardiac injury had lower lymphocyte counts and higher levels of inflammatory biomarkers compared with those without cardiac injury.^[27]^ Studies by D’Alto et al^[22,24,27,29,32,34,47,48]^ also found significant differences in myocardial enzyme levels in nonsurvivors compared with survivors, suggesting that these enzyme levels may have prognostic potential and affect pooled effect estimates. Therefore, the present work did not find a statistically significant difference; this may be related to the limited number of patients and the varying severity of the disease cases that were included in this meta-analysis.

Furthermore, obese patients may suffer from obstructive sleep apnea hypopnea syndrome, in which the body is chronically deprived of oxygen for a long time, leading to cardiopulmonary damage. When these patients are infected with SARS-CoV-2, they are likely to develop heart and respiratory failure, which can lead to worsening of the illness and even death. Similarly, obesity is a risk factor for diabetes mellitus, which is reportedly associated with an increased risk of adverse outcome events in patients with COVID-19.^[49]^ It has also been suggested that novel blood pressure and pulse pressure estimation based on pulse transit time and stroke volume approximation may better enable ubiquitous monitoring of blood pressure and management of cardiovascular risk indicators such as hypertension.^[50]^ Therefore, these indicators are likely to be used as part of a risk prediction model to predict the prognosis of COVID-19 patients.

Previous meta-analyses have concluded that TAPSE is lower in nonsurvivors compared with survivors.^[51]^ However, our study analyzed TAPSE not only in relation to mortality, but also in relation to RVD and cardiac injury. At the same time, TAPSE is a good bedside method for judging right heart function, and also a good guide for treatment. We also concluded that the relationship between TAPSE and COVID-19 patients was affected by disease severity. Our results further complement previous findings of an impact of hypertension and diabetes on mortality. We also discuss why TAPSE was chosen as a predictor and what factors have the opportunity to be part of a predictive model.

## 5 Limitations

There were several limitations to this study. Only English-language sources were included, excluding potentially eligible studies that were not written in English. Since this index measured by different echocardiographers may lead to deviations in the results, echocardiographers should be trained to unify the measurement standards and a larger sample size is needed for research. In addition, because of the different factors and outcomes investigated in each study, we were not able to comprehensively analyze only 1 factor. Finally, although we performed a subgroup analysis of TAPSE, a robust risk prediction model is required to accurately predict the prognosis of patients with COVID-19.

## 6 Conclusion

Low TAPSE levels are associated with poor COVID-19 disease outcomes. TAPSE levels are modulated by disease severity, and their prognostic utility may be skewed by pre-existing patient comorbidities.

## Author contributions

**Conceptualization:** Ye Tian, Huaihai Lu, Pei Zhang.

**Data curation:** Ye Tian, Huaihai Lu, Xuefang Liu, Yinlong Zhao.

**Formal analysis:** Ye Tian, Huaihai Lu.

**Funding acquisition:** Ye Tian, Huaihai Lu.

**Investigation:** Ye Tian, Huaihai Lu, Pei Zhang.

**Methodology:** Ye Tian, Huaihai Lu.

**Project administration:** Ye Tian, Huaihai Lu.

**Resources:** Ye Tian, Huaihai Lu.

**Software:** Ye Tian, Huaihai Lu.

**Supervision:** Ye Tian, Huaihai Lu, Pei Zhang.

**Validation:** Ye Tian, Huaihai Lu.

**Visualization:** Ye Tian, Huaihai Lu.

**Writing – original draft:** Ye Tian, Huaihai Lu.

**Writing – review & editing:** Ye Tian, Huaihai Lu, Pei Zhang.

## Supplementary Material

Supplemental Digital Content
